# Why Do We Screen Newborn Infants for Cystic Fibrosis?

**DOI:** 10.3390/ijns6030056

**Published:** 2020-07-08

**Authors:** Jürg Barben, Kevin W. Southern

**Affiliations:** 1 Division of Paediatric Pulmonology & CF Centre, Children’s Hospital of Eastern Switzerland, 9006 St. Gallen, Switzerland; 2 Department of Women’s and Children’s Health, University of Liverpool, Alder Hey Children’s Hospital NHS Foundation Trust, Liverpool L12 2AP, UK

The introduction and widespread implementation of newborn bloodspot screening (NBS) for cystic fibrosis (CF) has offered earlier diagnosis and better outcomes for children with CF in many countries of the world. It represents a paradigm shift in the diagnostic pathway for these families. In contrast to a clinical diagnosis, infants are now referred for diagnostic testing after a positive NBS result and, apart from a small proportion who present with bowel obstruction (meconium ileus), CF infants have no or only minimal clinical manifestation of the disease in the early days of life. Clinical symptoms can appear over the first few weeks, for example, insufficient weight gain, fatty stools or salt loss syndrome, but are often insidious and difficult to recognise.

The introduction of NBS has enabled the provision of early appropriate treatment (pancreatic enzyme replacement therapy, fat-soluble vitamins, salt supplementation and antibiotics) to prevent manifestations of the disease. In the near future, early diagnosis will facilitate the prompt use of new cystic fibrosis transmembrane conductance regulator (CFTR) modulator therapies that correct the basic underlying molecular defect.

NBS for CF has been a global success but continues to raise questions with many varied approaches and the development of new technologies, in particular the ability to undertake extensive gene examination. It is still valid to ask many questions:

- What is the best protocol to achieve high sensitivity and specificity?
- Should extensive genetic analysis be part of this algorithm, which enables the identification of many more *CFTR* variants?
- How to evaluate and manage inconclusive cases with a borderline sweat test or CFTR variants with unclear clinical relevance?
- What is the optimal approach to inform and counsel the parents about the NBS results and inconclusive findings?

These questions are not easy to answer and require a balanced solution that reflects the local health care system and may appropriately result in different answers around the globe.

The aim of this series of articles was to compile the current state of knowledge on NBS for CF and the questions arising from it. Using the framework of the network of the Newborn Screening Working Group (NSWG) of the European CF Society (ECFS), we approached colleagues from all over the world to submit articles for peer review. On the initiative of the *International Journal of Newborn Screening* (IJNS), the opportunity arose to realize this project, and we would like to take this opportunity to thank the authors for their excellent contributions and the IJNS for their support and cooperation. We feel the resulting series of articles provides a state-of-the-art evaluation of the current status of NBS for CF and provides much insight into the questions above and a path to improve quality across the globe.

The history of newborn bloodspot screening for CF is recorded by Georges Travert and Mary and Anthony Heeley, all of whom played an important role in these early developments. They cover the early use of the immune-reactive trypsinogen (IRT) assay, the challenges they and others faced and how they were overcome [1].

Lutz Naehrlich describes how early diagnosis, multidisciplinary care and optimized and preventive treatments have improved the outlook for people with CF. From his position as Director of the European Registry, he is able to give a clear picture of the changing face of CF, and the direct impact of NBS on this landscape [2].

One of the major challenges in the field of NBS for CF has been the collection of robust and comparable data across countries and regions. New Zealand was the first country to establish NBS for CF and Natasha Heather and Dianne Webster are well placed to reflect on the importance and challenges of collecting the correct metrics [3]. They highlight the critical importance of this if the quality of this public health initiative is to improve.

Virginie Scotet, Hector Gutierrez and Philip M. Farrell give an overview about the current situation of NBS for CF across the globe [4]. Each region has typically undertaken CF NBS after analysis of the advantages, costs and challenges, particularly regarding the relationship of benefits to risks. The review describes the lessons learned from the journey toward universal screening wherever CF is prevalent and an analytical framework for application in those undecided regions.

This complements the next article, in which Rachel Armstrong, Lucy Frith, Fiona Ulph and Kevin Southern consider NBS for CF from a bioethical perspective [5]. They report in detail all possible outcomes from NBS for CF and place these in an ethical framework. Placing these in the context of the genetic profile of the population screened, the geography of the region and the healthcare resources available, they propose an approach engaging with stakeholders to determine the best protocol for a region. 

Olaf Sommerburg and Jutta Hammermann describe in their review the strengths and weaknesses of pancreatitis-associated protein (PAP) in the algorithm of NBS for CF [6]. This biochemical test has emerged as an adjunct to IRT measurement, but the relationship is complex and is reviewed in detail by these authors who have considerable experience through implementing this assay as part of the protocol in Germany.

Anne Bergougnoux, Maureen Lopez and Emmanuelle Girodon give a summary of the role of DNA analysis in the CF screening programme. Their work in the national French laboratory gives them a unique insight into the challenges of incorporating genetic testing, especially extended gene analysis (EGA) [7].

A consequence of NBS for CF is the identification of infants with a positive screening test but an inconclusive diagnostic testing. Anne Munck led the European consensus exercise to better define the evaluation and management of these infants, in addition to leading the French research project that monitored outcomes. She places these results in the context of other work from around the globe [8].

The processing of a positive NBS result for CF not only consists of the screening part in the laboratory but also the interface between the family and healthcare, and ultimately the CF team. This is a complex process reviewed by Jürg Barben and Jane Chudleigh, both of whom have undertaken extensive research projects examining these issues [9]. It is clear that this is an area that needs considerable improvement across the globe and the authors review evidence of good practice and propose a roadmap to improve the quality of this difficult process.

Consistent with the article above is a detailed review of the psychological impact of NBS for CF by Jane Chudleigh and Holly Chinnery [10]. A better understanding of the journey that the families of infants with a positive NBS result go on enables CF teams to predict and ameliorate unnecessary distress.

Again we thank all the authors; there is much to celebrate in the field of NBS for CF, but clearly still work to do, and this experienced faculty of authors has provided a series of state-of-the-art articles to help achieve that goal. In addition, we would like to thank the 19 experts who provided high-quality peer review (sometimes twice) for this series. We were extremely grateful for their comprehensive and timely contributions, which were important for the overall quality of the series.
